# The complete mitochondrial genome of the copepod *Calanus glacialis*

**DOI:** 10.1080/23802359.2017.1361357

**Published:** 2017-08-06

**Authors:** Marvin Choquet, Homère J. Alves Monteiro, Johan Bengtsson-Palme, Galice Hoarau

**Affiliations:** a Faculty of Biosciences and Aquaculture, Nord University, Bodø, Norway;; b Department of Infectious Diseases, Institute of Biomedicine, The Sahlgrenska Academy, University of Gothenburg, Gothenburg, Sweden

**Keywords:** *Calanus glacialis*, copepod, mitochondrion, mitogenome, zooplankton

## Abstract

*Calanus glacialis*, a marine planktonic copepod, is a keystone species in the Arctic Ocean. In this study, we shotgun sequenced the total DNA of one *C. glacialis* individual, using the NextSeq^®^ Illumina platform, in order to determine its mitochondrial genome sequence. We successfully assembled and annotated this 20,674 bp long sequence, which included 13 protein-coding genes, 2 rRNA genes and 22 tRNA genes. Common gene-coding regions of 19 other species were used to reconstruct a phylogenetic tree, using mitogenomes of the phylogenetically closest copepods available. The new resource described here constitutes a tool of interest for better understanding the structure and dynamics of *C. glacialis* populations.

The genus *Calanus* consists of 26 distinct marine copepod species (WoRMS Editorial Board, 2017), present in every ocean in the world as part of the zooplankton. Despite their ecological importance, only two mitochondrial genomes have been reported within the *Calanus* genus: *C. sinicus* (Minxiao et al. 2011) and *C. hyperboreus* (Kim et al. 2013). *C. glacialis* is one of the key species of the Arctic Ocean, as the crucial link between primary production and higher trophic levels such as fishes, invertebrates, marine mammals and birds (Falk-Petersen et al. 1990).

In this study, we report the complete sequence of the mitochondrial genome of *C. glacialis*. We selected one *C. glacialis* individual from Sørfolda (Norwegian coast: 67°30 N, 15°28 E), which we identified as such using a set of nuclear molecular markers (Smolina et al. 2014). Total DNA was extracted using the E.Z.N.A.^®^ Insect DNA Kit and was shotgun sequenced on the NextSeq^®^ Illumina platform. Given the amount of DNA recovered from a single individual, everything was used for the library construction*. De novo* assembly of the filtered reads was performed using Ray version 2.3.1 (Boisvert et al. 2010) with a k-mer length of 31. Contigs that matched the mitochondrial genomes of *C. hyperboreus* or *C. sinicus* in a BLAST (Altschul et al. 1997) search (e-value cut-off 10^−10^) were extracted. To potentially further merge these contigs, they were used as seeds in a Peacat search (http://microbiology.se/sw/petkit) against all assembled contigs (Bengtsson-Palme et al. 2014). The resulting consensus sequences were tested for circularity using Pemap (http://microbiology.se/software/petkit/), but no evidence of circularity was found.

We mapped the annotated mitochondrial genomes of *C. hyperboreus* and *C. sinicus* to the longest contig obtained from the assembly and were able to identify all expected mitochondrial genes.

The mitochondrial sequence of *C. glacialis* is 20,674 bp long and contains 13 protein-coding genes (total of 3458 amino acids), 2 rRNA genes, 22 tRNA genes and 1 putative control region. The sequence is composed of 31.7% base A, 28.8% base T, 19.6% base C and 19.9% base G. Ribosomal 12S and 16S RNA are 656 bp and 1138 bp long, respectively. The sequence has been deposited in GenBank under the accession number MF422146.

A phylogenetic analysis was performed using all coding genes for 18 species of Crustaceans (including 10 copepods species) and two hexapods as out-groups (*Japyx solifugus* and *Campodea fragilis)*. The phylogenetic tree was reconstructed with a maximum likelihood method using PHYML (Guindon and Gascuel 2003) (GRT + I + G model, 1000 bootstraps) (Figure 1). All copepods formed a monophyletic group and *C. glacialis* clustered with the other two *Calanus* mitogenome (100% support). Given the ecological importance of *C. glacialis* within the Arctic ecosystem, the newly determined mitogenome will be useful for investigating the history of *C. glacialis* populations and their spatiotemporal variability.

**Figure 1.**
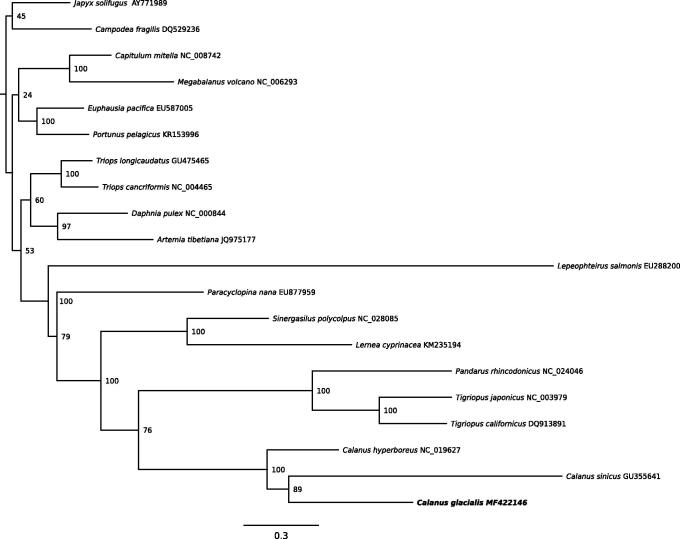
Phylogenetic tree of *C. glacialis* and 19 other species, with *Japyx solifugus* and *Campodea fragilis* as out-groups. ML bootstrap values (1000 replications) are indicated in front of each node.
